# Severe purulent pericarditis caused by invasive *Eikenella corrodens*: case report and literature review

**DOI:** 10.1186/s12879-019-4256-0

**Published:** 2019-07-23

**Authors:** Wei Wei, Hu Nie

**Affiliations:** Emergency Department of West China Hospital, Sichuan University, Chengdu, China

**Keywords:** *Eikenella corrodens*, Infection, Pericarditis

## Abstract

**Background:**

*Eikenella corrodens* is a slowly growing gram-negative bacillus that can cause severe invasive disease in human. Although E. corrodens infections in various sites of human body have been well described, pericarditis caused by invasive E. corrodens has rarely been reported.

**Case presentation:**

Here we report the case of a 63-year old male with a complaint of left shoulder pain. The patient was diagnosed as purulent pericarditis by chest computed tomography scan and ultrasound-guided pericardiocentesis, and the pathogen of E. corrodens was identified in the pericardial fluid culture. The clinical condition of the patient deteriorated quickly, and he died right after the drainage surgery even though the pathogen was sensitive to antibiotics treatment.

**Conclusion:**

E. corrodens is a rare pericarditis associated pathogen. Purulent pericarditis caused by E. corrodens presents atypical manifestations and rapid progression of infection in immunosuppressed individuals such as neutropenic patients. Earlier diagnosis and proper drainage surgery with effective antibiotics treatment may improve the prognosis.

## Background

*Eikenella corrodens*, a small fastidious, facultative, anaerobic gram-negative bacillus, normally inhabit in dental plaque [1]. Although it is an uncommon cause of infection, its pathogenic potential has been recognized. E. corrodens could independently cause serious infection in both immunocompetent and immunocompromised hosts. Diseases caused by this pathogen include periodontitis, sinusitis, bite wound infections, head and neck infection, respiratory tract infections, abdominal infections, gynecologic infections, meningitis, spinal infection, endocarditis, and osteomyelitis [2–9]. Herein we report a case of purulent pericarditis caused by E corrodens and we also review the literature on E corrodens infections.

## Case presentation

A 63-year-old male was admitted to the emergency department with a complaint of left shoulder pain for one month and chest tightness for 3 days. The patient had a history of diabetes mellitus and his glucose was well regulated. Six years prior the patient had been treated with esophagectomy and radiotherapy for esophageal cancer. After the surgery, he had 3 times of following-up gastroscopy examinations, which indicated no evidence of recurrence, while the white blood cell count (WBC) kept in relatively lower level around 3.5 × 10^9^ /L. At admission he had no cough, vomiting and abdominal pain.

The patient had a temperature of 37.6 °C, a blood pressure of 123/87 mmHg, a pulse of 103 bpm, a respiratory rate of 20 per minute and an oxygen saturation of 97% at admission. There was no paradoxical pulse. His jugular veins were slightly distended. Lung auscultation revealed crackles at both lung bases, while the heart sounds were not distant. There was mild edema in both legs.

**Fig. 1:**
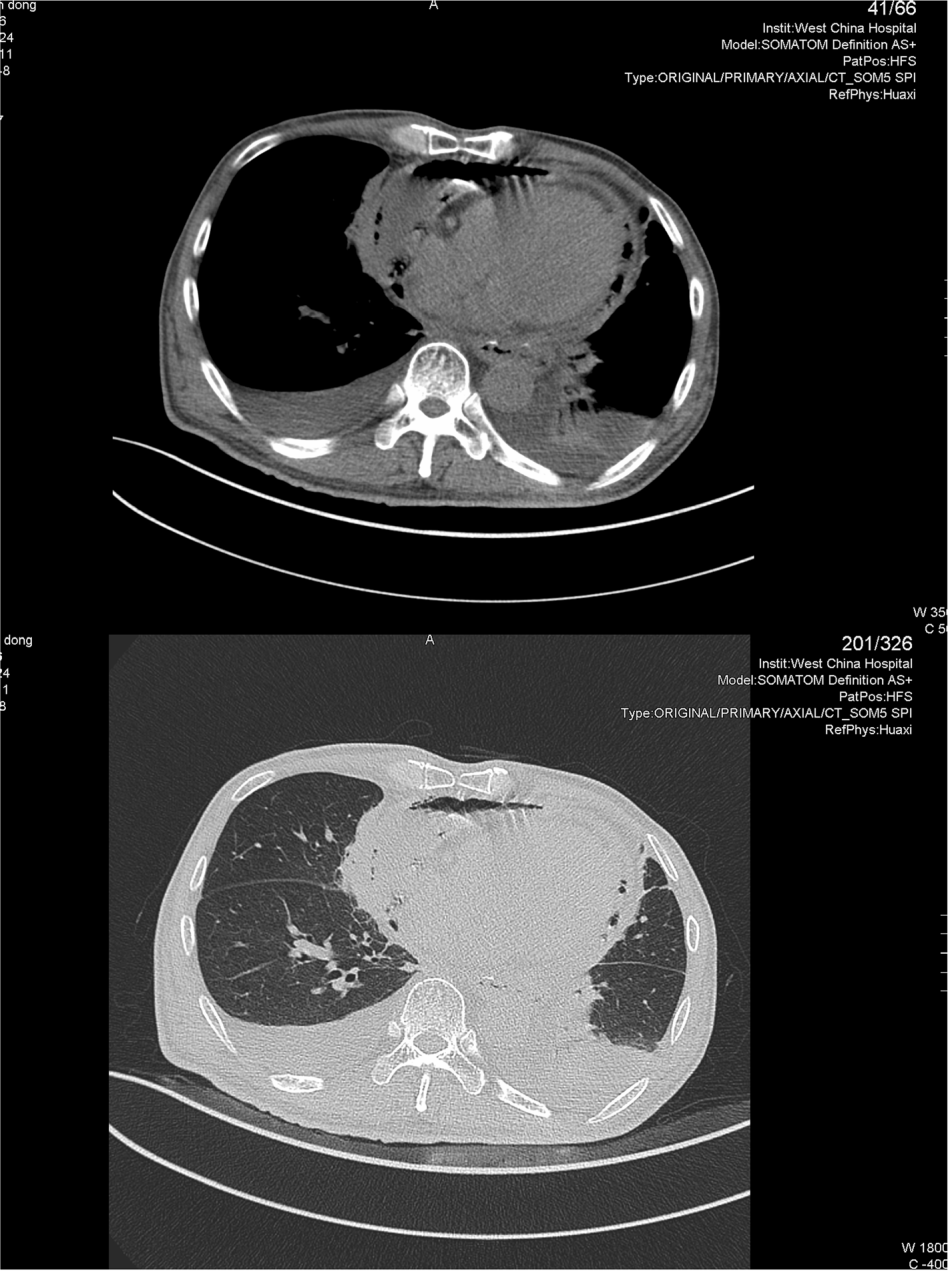
CT scan of the thorax at mid-thoracic level showing widened mediastinum, pyopneumopericardium, bilateral pleural effusion, and collapse of the lower lobe of left lung

The laboratory examination indicated WBC of 12 × 10^9^/L and serum procalcitonin level of 1.8 ng/ml, while the liver function tests and serum myocardial markers level (troponin T and pro-b-type natriuretic peptide) were slightly above normal. Chest computed tomography revealed a massive pyopneumopericardium, a bilateral pleural effusion, and a collapse of the lower lobe of left lung (Fig. 1). Ultrasound-guided pericardiocentesis was performed and 300 ml of pus was drained. Analysis of specimens of pericardium pus indicated WBC of 20 × 10^9^/L (100% neut), lactic dehydrogenase (LDH) > 17,000 IU/L and glucose of 0.05 mmol/L.

Parenteral treatment with ceftriaxone 2000 mg IV every 24 h was given at admission, but no clinical improvement was achieved. On the day after his admission the pericardial fluid culture indentified E. corrodens and *Streptococcus anginosus*, and repeated blood test indicated increased WBC of 28 × 10^9^/L and extremely abnormal blood biochemical indexes including alanine aminotransferase (ALT) of 5,000 IU/L, aspartate aminotransferase (AST) > 10,000 IU/L, LDH of 10,000 IU/L, amylase of 366 IU/L, and hypoalbuminemia of 25 g/L. Serum myocardial markers levels increased dramatically (Troponin T of 198 ng/L and pro-b-type natriuretic peptide of 3,000 pg/ml). Arterial blood gas analysis revealed apparent acidosis and type I respiratory failure (oxygen partial pressure of 55 mmHg, lactate of 9.8 mmol/L). The patient’s vital signs worsened on day 2 after admission with blood pressure of 84/45 mmHg, pulse of 154 bpm, respiratory rate of 42 per minute and oxygen saturation of 78%. The antibiotic therapy was changed to imipenem 500 mg IV every 8 h, and mechanical ventilation was provided. But the patient’s condition still had no improvement. The antimicrobial susceptibility test showed that the isolated strain was sensitive to imipenem and ceftriaxone, while resistant to clindamycin and amykacin. The patient died three days after admission.

## Discussion and conclusion

*Eikenella corrodens* is a ubiquitous bacillus of the oral flora. Belonging to the HACEK bacteria group (Haemophilus species, Aggregatibacter species, Cardiobacterium hominis, *Eikenella corrodens*, and Kingella species), E. corrodens has been shown to cause endocarditis, mediastinitis or pleuropulmonary infections in the thoracic cavity [10–13]. Pericarditis caused by E. corrodens is rare. Only four cases of pericarditis caused by E. corrodens were indentified in a Medline literature search (Table 1). In these cases, the patients were immunocompetent without any evidence of local lesions such as oesophageal perforation. Peritonsillar infection occurred in two cases, and it was suspected as primary sources. The antimicrobial sensitivity and susceptibility test indicated that E. corrodens were commonly resistant to clindamycin but sensitive to ampicillin. With prolonged treatment in these 4 cases reported, the patients had a good prognosis [14–17].

**Table 1:** Clinical features of three patients with pericarditis due to *Eikenella corrodens*

Case No. [Reference]	Age/Sex	Underlying condition	Other associated organisms	Associated infection	Medical treatment (duration)	Clinical context	Outcome	Sensitive	Resistant
1 [14]	28/female	Minimal bronchiectasis	no	mediastinitis, pneumonia	Amoxycillin (3 weeks)	Pericardiotomy and upper mediastinal drain	Cured	Ampicillin Gentamicin Cefuroxime	Clindamycin
2 [15]	52/male	no	Haemolytic streptococci of group A	Peritonsillar abscesses pleural effusion	Ticarcillin (3 weeks)	Pericardiocentesis Pleural drainage	Cured	Unknown	Clindamycin Amikacin
3 [16]	29/female	Tonsillectomy	no	pneumonia, pleuritis, pleural effusion, cholecystitis	Moxifloxacin (3 weeks) Ceftriaxone (4 weeks)	Thoracotomy Pericardiocentesis	Cured	Unknown	Unknown
4 [17]	32/male	Bronchomediastinal fistula	no	Bilateral pleural effusions	Ampicillin (4 weeks)	Thoracocentesis Operative drainage of the pericardial sac Debridement of the left lung, silicone stent placement at the left main bronchus	Cured	Ampicillin	Unknown
This case	63/male	Diabetes mellitus esophageal cancer with surgery and radiotherapy	*Streptococcus anginosus*	pneumonia pleuritis pleural effusion	Imipenem (2 days)	Pericardiocentesis	Died	Imipenem Ceftriaxone	Clindamycin Amikacin

In the case reported here, the port of entry could not be determined. But the past esophageal cancer treated with surgery and radiotherapy and diabetes mellitus may be the important risk factors. The relatively lower level of WBC indicated the patient may be immunocompromised for a long time. Patients with neutropenia may present atypical manifestations of infection, rapid progression of infection, unusual infecting organisms, and unusual sites of involvement [18].

The *S. anginosus* and E. corrodens, both found in the oral cavity and the gastrointestinal tract may cause localized suppurative infections in different body sites. The interaction between these microorganisms enables them to disrupt the mucosal barrier and invade deeper tissues, which leads to mixed infections, especially in infectious processes with host defenses compromised [19].

According to the antimicrobial sensitivity and susceptibility test, the empirical antibiotic treatment for the patient should be effective. However, the clinical condition of the patient deteriorated quickly after the drainage indicated that the diagnosis, pericardiocentesis and treatment was initiated too late or not at the right time, and the surgery may spread the infection without enough or strong antibiotics treatment previously. Unfortunately, further chest radiography was not obtained to confirm the hypothesis. On the other hand, the unfavorable outcome could be related to the patient’s later age, to the neutropenia induced by recurrent tumors treated with radio and chemotherapy. Furthermore, we inferred that patient’s atypical symptoms contributed to the delay in seeking medical service and rapid progression of infection.

The case reported here presents a rare pericarditis caused by E. corrodens. In immunosuppressed host E. corrodens is an important pathogen associated with a spectrum of intrathoracic suppurative infections by itself or as part of mixed flora. Earlier diagnosis and proper drainage surgery with effective antibiotics treatment may improve the prognosis.

## Data Availability

The dataset of this article will not be available publicly, to ensure the patient’s’ privacy, but are available from the corresponding author on reasonable request.

## Abbreviations

ALT: Alanine aminotransferase
AST: Aspartate aminotransferase
LDH: Lactatdehydrogenase
